# Transarterial chemoembolization combined with computed tomography-guided ^125^iodine implantation enhances survival in hepatocellular carcinoma patients with portal vein tumor thrombus

**DOI:** 10.18632/oncotarget.16491

**Published:** 2017-03-23

**Authors:** Hong Tao Hu, Jun Peng Luo, Hai-Liang Li, Chen Yang Guo, Quan Jun Yao, Xiang Geng, Li Jiang

**Affiliations:** ^1^ Department of Radiology and Research Institute of Radiology, The Affiliated Cancer Hospital of Zhengzhou University, Henan Cancer Hospital, Zhengzhou, Henan Province 450003, P. R. China

**Keywords:** hepatocellular carcinoma, portal vein tumor thrombus, transarterial chemoembolization, ^125^iodine implantation, computed tomography-guided

## Abstract

We conducted a retrospective study to evaluate the safety and efficacy of transarterial chemoembolization (TACE) combined with computed tomography-guided ^125^iodine implantation (TACE-^125^iodine) in hepatocellular carcinoma (HCC) patients with type B portal vein tumor thrombus (PVTT). From medical records, we determined that 50 patients who received ^125^iodine implantation 4-7 days after the first TACE session showed better survival than 50 patients who received only TACE (median survival, 13.1 vs. 6.0 months; *P*<0.01). Moreover, the PVTT control rate was higher in the TACE-^125^iodine than TACE alone group (78% vs. 18%; *P*<0.01). Multivariate analysis demonstrated that the TACE-^125^iodine procedure was an independent prognostic factor for overall survival. We also observed that bilirubin levels were increased at 4 weeks, indicating that ^125^iodine seeding in the PVTT beneficially impacted the small bile duct, which is proximal to the portal vein. No severe adverse events were observed in patients that received ^125^iodine seed implantation, and the mild adverse events were successfully treated. This study shows that TACE-^125^iodine therapy enhances patient survival with minimal adverse events. It is also more affordable than sorafenib, which is currently the recommended therapy for advanced HCC patients with PVTT.

## INTRODUCTION

Hepatocellular carcinoma (HCC) ranks fifth among the most common cancer globally and is the third most frequent cause of cancer deaths [1–2]. A common complication of HCC is the Portal vein tumor thrombus (PVTT) that results in a poor median survival of 2.7–4.0 months and is reported in 64.7% cases at autopsy [3–6]. The poor prognosis associated with PVTT is due to increased risk of tumor spread; variceal hemorrhage as a result of increased portal venous blood pressure; and complications including ascites, jaundice, hepatic encephalopathy, and liver failure due to decreased portal flow [7, 8]. Further, since PVTT is a contraindicator for transplantation and curative resection according to the HCC treatment guidelines, the treatment options are limited [9–10]. Therefore, the standard management for HCC patients with PVTT is controversial [8, 10, 11].

The efficacy of sorafenib, which is the recommended therapy for HCC patients with PVTT according to the Barcelona Clinic Liver Cancer staging system, is limited [12–13]. However, patients with PVTT that underwent transarterial chemoembolization (TACE) in addition to sorafenib demonstrated better survival according to some studies [14–15]. Therefore, TACE therapy is considered for advanced-stage HCC patients [1, 16–17]. However, since sorafenib is expensive, the majority of patients in China with advanced HCC (with PVTT) cannot afford such treatment, and therefore, there is an urgent need for an alternative and more cost-effective treatment.

Yang *et al*. reported that TACE in combination with endovascular implantation of a ^125^iodine seed strand was a feasible and effective treatment for HCC patients with PVTT [19]. Some studies reported computed tomography (CT)-guided ^125^iodine implantation in HCC patients with PVTT [20–21]. The two methods are different, and direct CT-guided ^125^iodine implantation in PVTT is technically easier to perform. However, these studies included very few cases of CT-guided ^125^iodine seed implantation in PVTT without any control group or analysis of long-term outcomes.

Thus, we evaluated the safety and efficacy of TACE combined with direct CT-guided ^125^iodine implantation in PVTT (TACE-^125^iodine) in a retrospective cohort study of HCC patients that had PVTT only in the right or left portal vein branch and absent in the main portal vein.

## RESULTS

### Patient characteristics

**Figure 1:**
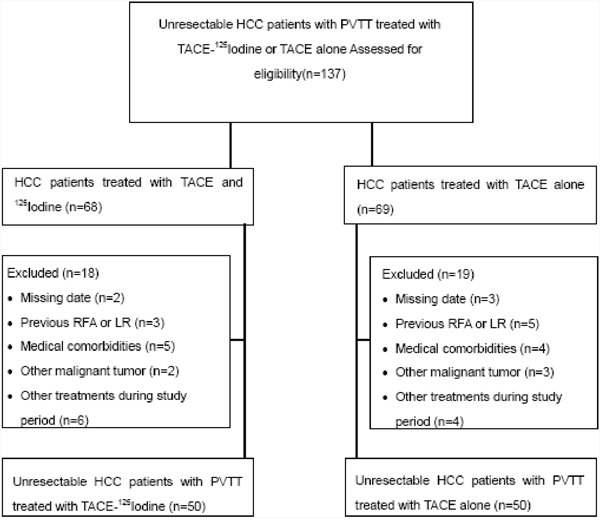
Flow diagram shows exclusion citeria

Out of 137 patients with unresectable HCC with type B PVTT that underwent either TACE-^125^iodine or TACE only, 37 were excluded because they did not meet the inclusion criteria (Figure 1). As a result, we studied 100 patients (TACE-^125^iodine, n=50; TACE only, n=50) that qualified for our analysis. Detailed baseline patient characteristics are shown in Table 1. The baseline characteristics between the TACE-^125^iodine and the TACE only groups were not statistically different. The mean follow-up duration for the TACE-^125^iodine group was 24.2 months (range, ≤1 month to 56 months) and 6.3 months (range, ≤1 month to 18 months) for the TACE only group. During the follow-up, 44 of the 50 patients (88%) in the TACE-^125^iodine group and 49 of the 50 patients (98%) in the TACE only group died. Five (10%) of the 50 patients underwent repeated ^125^iodine seed implantation with a mean average of 1.3 (range, 1–2) procedures per patient in the TACE-^125^iodine group. Thirty-six of the 50 patients (78%) in the TACE-^125^iodine group and 33 of the 50 patients (73%) in the TACE only group underwent repeated TACE, with a mean average of 3.6 (range, 1–8) and 3.1 (range, 1–6), respectively (Table 2). The bilirubin level increased at 4 weeks after ^125^iodine seed implantation but recovered to baseline values at 8 weeks (Table 3 and Table 4).

**Table 1:** Baseline Patient Characteristics

Characteristic	TACE	TACE-^125^I	*P* Value
Sex			.603*
Male	40(80)	42(84)	
Female	10(20)	8(16)	
Age(y)^†^	45.4±5.2	47.6±6.3	.321^‡^
ECOG performance			.689*
0	25(50)	27(54)	
1-2	25(50)	23(46)	
Cause of liver disease			.455*
Hepatitis B	40(80)	42(84)	
Hepatitis C	8(16)	7(14)	
Other	2(4)	1(2)	
Child-Pugh score			.564*
A	44(88)	42(84)	
B	6(12)	8(16)	
Morphology			.641*
Massive	27(54)	26(52)	
Multi-nodular	23(46)	24(48)	
Maximum diameter			.638^‡^
>5cm	31(62)	30(60)	
≤5 cm	19(38)	20(40)	
Baseline laboratory test result^†^			
Total bilirubin level(μmol/L)	22.1±3.4	21.3±5.2	.576^‡^
Albumin level (g/L)	36.4±2.3	35.2±3.1	.645^‡^
α-Fetoprotein level (ng/mL)			.688*
>400	28(56)	26(52)	
≤400	22(44)	24(48)	
Extrahepatic spread			.309*
Lymph nodes	10(20)	12(25)	
Lung	5(10)	4(8)	
Boes	2(4)	2(4)	
Suprarenal gland	1(2)	1(2)	

Note: Unless indicated, date are numbers of patients, and numbers in parentheses are percentages.

ECOG=Eastern Cooperative Oncology Group.

* Pearson χ^2^ test was used.

†Date are mean±standard deviation.

‡Independent-samples *t* test was used.

**Table 2:** Times of TACE procedures per patient in two groups

Treatment method	No. of Patients(*n*=100)	Times of TACE	*P* Value*
TACE	50	3.6±0.5	0.658
TACE-^125^I	50	3.1±1.1	

Note: Date in parentheses are 95%CIs. ECOG=Eastern Cooperative Oncology Group.

* Independent-samples *t* test was used.

**Table 3:** Liver Function Changes 4 Weeks after Treatment in the TACE-^125^I Group

Liver Function Test	Pretreatment	4 Weeks after Treatment	*P* Value*
Total bilirubin level(μmol/L)	21.3±5.2	42.5±3.5	.023
Albumin level (g/L)	35.2±3.1	36.1±2.1	.053
Prothrombin time (sec)	12.8±0.5	13.2±0.4	.062

Note: Unless indicated, date are mean±standard deviation.

*Paired-Samples T test was used

**Table 4:** Liver Function Changes 8 Weeks after Treatment in the TACE-^125^I Group

Liver Function Test	Pretreatment	8 Weeks after Treatment	*P* Value*
Total bilirubin level(μmol/L)	21.3±5.2	22.0±0.5	.623
Albumin level (g/L)	35.2±3.1	37.1±1.1	.453
Prothrombin time(sec)	12.8±0.5	12.8±1.4	.762

Note: Unless indicated, date are mean±standard deviation.

*Paired-Samples T test was used

### Adverse events related to TACE

Minor TACE-related adverse events like new ascites, liver dysfunction, fever, abdominal pain, and nausea or vomiting were reported in most patients of both groups. The adverse events were treated symptomatically in the hospital, and all patients recovered from the events. Severe adverse events such as spontaneous bacterial peritonitis, gastrointestinal hemorrhage, liver abscess, or pulmonary or cerebral oil embolization were not encountered during or after the TACE procedure in any patient.

### Adverse events related to ^125^iodine seed implantation

Few acute adverse events were reported due to ^125^iodine seed implantation. Three patients had pneumothorax with collapsed lung (<30% of the ipsilateral lung), but recovered after conservative treatment. In four cases, minor subcapsular liver hemorrhage was observed, which was treated conservatively with success. In two cases, seed floatation to the normal liver parenchyma was observed that did not require any medical management. Apart from these, other severe adverse events such as hemorrhage, bile fistula, abscess formation, acute peritonitis, and liver failure, were not observed in relation to^125^iodine seed implantation.

### Intrahepatic lesions and PVTT response

During the last follow-up, two experienced radiologists (HLL and CYG) evaluated the intrahepatic lesions response rate according to the mRECIST assessment of HCC [25]. The intrahepatic lesions control rate was 86% for the TACE-^125^iodine group and 88% for the TACE alone group. The intrahepatic lesions responses are shown in Table 5. There were no significant differences among the two groups in relation to the objective response rate of intrahepatic lesions (*P=0.766*).

**Table 5:** Intrahepatic Lesions Responses for the Two Groups

Intrahepatic lesions responses	TACE-^125^I	TACE-alone	*P* Value
Total patients	50	50	
Complete response	0	0	
Partial response	34	36	
Stable disease	9	8	
Progressive disease	7	6	
Disease control rate (%)	86	88	0.766

Note: Disease control rate (DCR) was calculated with the following equation: DCR = (SD + PR + CR)/N, where SD is number of patients with stable disease, PR is number of patients with partial response, and CR is number of patients with complete response. P values<.005 indicated a significant difference (Pearson Chi-Square test).

A new system was used to categorize the PVTT responses based on CT scans or MRI. The categories were as follows: (1) Complete response (CR) = PVTT disappearance and restored portal vein blood flow; (2) Partial response (PR) = PVTT disappearance or a >30% reduction in thrombus in the greatest cross-sectional area and improved portal vein blood flow; (3) Stable disease (SD) = <30% reduction in thrombus in the greatest cross-sectional area; and (4) Progressive disease (PD) = extended thrombus [19]. The PVTT responses are shown in Table 6. The PVTT control rate for the TACE-^125^iodine group was 78% compared to 18% in the TACE only group (*P*<0.01).

**Table 6:** Tumor Responses in PVTT for the Two Groups

Tumor Response in PVTT	TACE-^125^I	TACE-alone	*P* Value
Total patients	50	50	
Complete response	0	0	
Partial response	20	1	
Stable disease	19	8	
Progressive disease	11	41	
Disease control rate (%)	78	18	<.001

Note: Disease control rate (DCR) was calculated with the following equation: DCR = (SD + PR + CR)/N, where SD is number of patients with stable disease, PR is number of patients with partial response, and CR is number of patients with complete response. P values<.005 indicated a significant difference (Pearson Chi-Square test).

**Figure 2:**
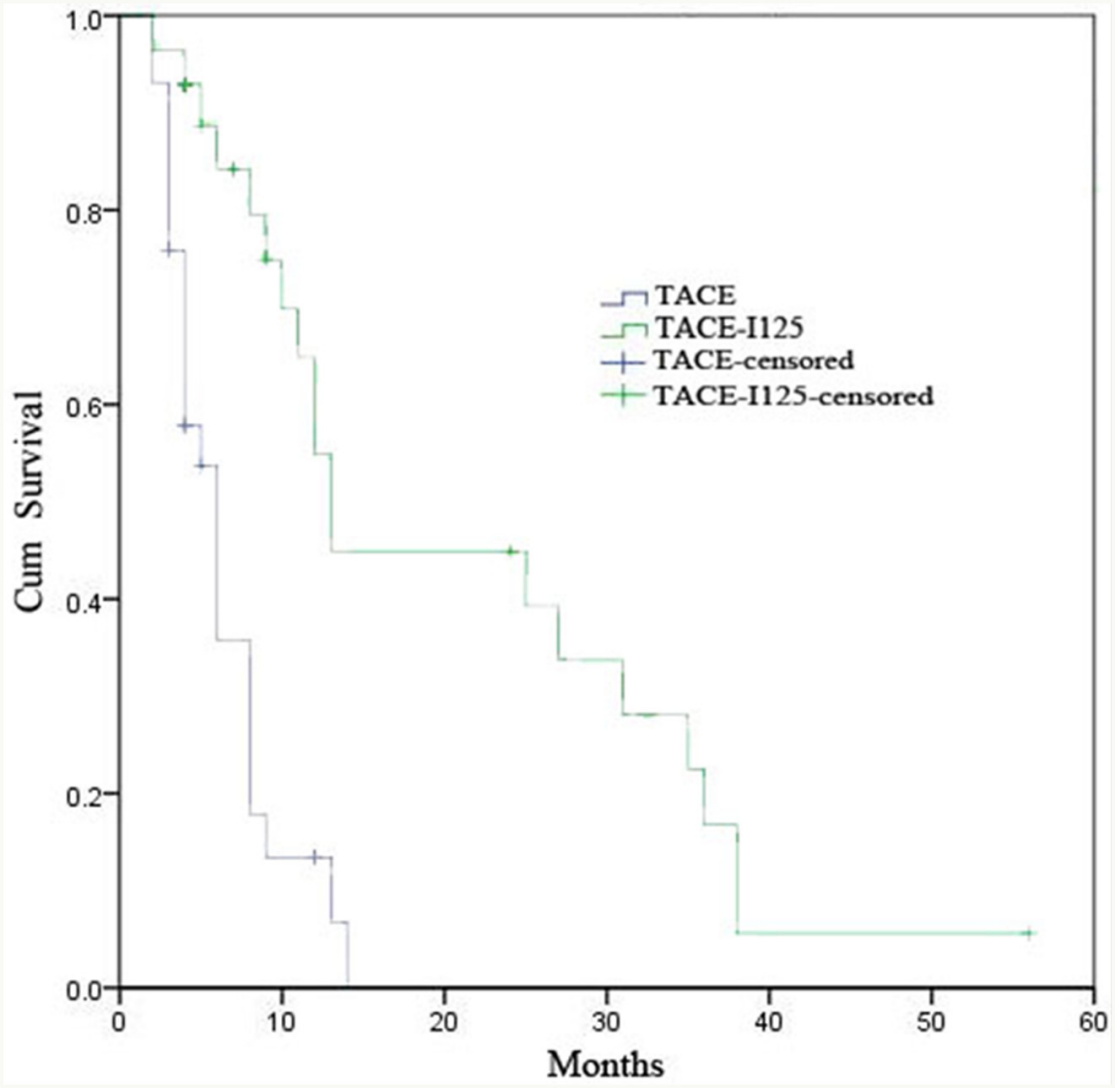
Kaplan-Melier cureves of OS in patients with HCC and PVTT who underwent TACE-125iodine or TACE

The median OS in the TACE-^125^iodine group was 13.1 months (95% confidence interval [CI]: 10.1-15.1) and 6.0 months (95% CI: 4.3-7.7) in the TACE only group (*P*<0.01; Figure 2). Univariate analyses revealed that patients undergoing TACE-^125^iodine treatment demonstrated better OS than the TACE only patients (Table 7; *P*<0.01). On the basis of these findings, multivariate analysis demonstrated that treatment method was an independent prognostic factor for OS (Table 8).

**Table 7:** Univariate Analysis of Prognostic Factor for OS

Factor	No. of Patients(*n*=100)	Median OS(mo)	*P* Value*
ECOG performance			.532
0	46	8.2(4.7,12.1)	
1-2	54	8.3(6.8,9.6)	
Child-Pugh score			.653
A	75	8.3(7.2,9.8)	
B	25	8.3(5.6,11.6)	
Treatment method			<.01
TACE	50	6.0(4.3,7.7)	
TACE-^125^I	50	13.0(10.1,15.1)	
α-Fetoprotein level (ng/mL)			.687
>400	55	8.5(7.3,11.3)	
≤400	45	8.6(7.2,11.6)	

Note: Date in parentheses are 95%CIs. ECOG=Eastern Cooperative Oncology Group.

* Log-rank test was used

**Table 8:** Multivariate Analysis of Prognostic Factor for OS

Factor	Hazard Ratio^#^	*P* Value*
Treatment method		<.001
TACE	2.54(1.52,3.75)	
TACE-^125^I	1	

Note: Date in parentheses are 95%CIs.

* Cox regression was used

## DISCUSSION

Song *et al*. reported that median OS in HCC patients with PVTT treated with hepatic arterial infusion chemotherapy or sorafenib was 7.1 and 5.5 months, respectively [26]. Furthermore, Tang *et al*. reported in a retrospective study consisting of 371 patients with resectable HCC with PVTT that the median OS was 10.0 and 12.3 months for surgical resection and conformal radiotherapy combined with TACE, respectively [27]. In comparison, this study demonstrated a median OS of 13.1 months in the TACE-^125^iodine group and 6.0 months in the TACE alone group, thus demonstrating a significant survival benefit of TACE-^125^iodine procedure in HCC patients complicated with PVTT in the right or left portal vein branches. This implied that TACE induced extensive intrahepatic tumor necrosis, whereas ^125^iodine implantation improved local tumor control in PVTT, which probably delayed PVTT progression thereby reducing the risk for tumor intrahepatic metastasis [28, 29]. This showed that TACE combined with ^125^iodine implantation could be used to effectively treat HCC patients with PVTT.

Similar to our results, HCC patients with PVTT that received conformal radiation therapy combined with TACE and hepatic arterial chemotherapy demonstrated response rates between 27.5%–75% with a median OS of 7–13 months [30, 31]. The advantages of implanting ^125^iodine seeds in the PVTT [21, 32] are as follows: (a) the radiation from seeds rendered a sustained and higher accumulative dose within the PVTT while causing minimal injury to the surrounding normal tissues, whereas serious complications like hepatitis have been reported in traditional; (b) therapy with ^125^iodine seeds had a low volume loss since it was not affected by respiratory movements; (c) the biological effect of the^125^iodine seeds was superior to three-dimensional conformal radiation since they eliminated tumor cells by maintaining them in a resting state and changing their immunophenotype thereby decreasing metastasis [30]; and this method shortened the hospitalization time of the patient thereby reducing the medical expenses when compared to conformal radiation therapy.

Similar to our data, Zhu *et al*. reported that HCC patients with type B PVTT of the first-order portal vein branch that underwent TACE combined with sorafenib had a median OS of 13.0 months [18]. Interestingly, all patients we analyzed underwent repeated TACE with the mean number of TACE procedures in the TACE-^125^iodine group being 3.1 (range, 2–6) and 3.6 (range, 2–8) in the TACE only group. Although the longer median OS in the TACE-^125^iodine group resulted in no significant difference in the mean number of TACE procedures between the groups (*P*=0.658), we observed that ^125^iodine implantation controlled PVTT more effectively and reduced intrahepatic metastasis. In addition, decreased intrahepatic metastasis reduced the need for the TACE procedure during the same period. Moreover, total bilirubin levels increased 4 weeks after ^125^iodine implantation and stabilized after 8 weeks indicating that ^125^iodine seeds beneficially impacted the small bile duct, which is proximal to the portal vein. Also, radiological cholangitis was mild and patients recovered without intervention.

Univariate and multivariate analysis showed that treatment method was the only independent pretreatment prognostic factor of OS. This implied that TACE combined with ^125^iodine seed implantation could be an effective and economical treatment option for HCC patients with PVTT in the right and left portal vein branches, especially for those who could not afford sorafenib. CT-guided ^125^iodine implantation was well tolerated generally with the most common adverse events being puncture site pain, seed transmigration, and a small degree of subcapsular hemorrhage that were manageable. Also, radiation-induced toxicities that are generally observed with external radiation such as loss of appetite, nausea, duodenal ulcer, hematologic abnormality, and gastrointestinal bleeding were not found [26–29]. Puncture site pain disappeared one or two days after the procedure and severe pain was treated orally by analgesic drug. Seed transmigration to the normal liver parenchyma resulted in only mild liver injury and was not addressed. The small degree of subcapsular hemorrhage that was detected on the CT scan was subsequently treated with abdominal bandage and intravenous hemostatic. Overall, severe adverse events were not observed in this study.

There are several limitations in the current study. First, this study was retrospective and therapeutic options (TACE-^125^iodine vs. TACE alone) in patients with advanced HCC with PVTT were individually determined on the basis of the attending physician's preference, which likely led to selection bias within the patient population. However, the bias was limited by choosing similar baseline characteristics between the two groups [18]. Second, the sample size in this study was small and from a single center thereby probably introducing inherent bias. Thus, large prospective and multi-center studies are necessary to evaluate the efficacy of TACE-^125^iodine treatment in HCC patients with PVTT.

In conclusion, our study shows that TACE-^125^iodine procedure in unresectable HCC patients with PVTT enhances survival with minimal adverse events and is more affordable than sorafenib.

## MATERIALS AND METHODS

### Study design and patient selection

We reviewed the electronic medical records of 137 patients with unresectable HCC and PVTT that underwent TACE only or TACE^125^iodine procedure at Henan Cancer Hospital of China from January 2009 to December 2011. This study was approved by the ethics committee of our institution. The retrospective analysis of data was approved by the institutional review board. Before the patients underwent treatment, the TACE only and TACE-^125^iodine treatment strategies were introduced to them by the attending physicians (YQJ and GX, who introduce the two treatment methods) and the final treatment strategy was decided by the patients. If the patients chose the TACE-^125^iodine method, CT-guided ^125^iodine (CIAE-6711; Chinese Atomic Energy Science Institution, Beijing) was implanted (4.8-mm long × 0.8-mm wide) in the PVTT 4–7 days after the first TACE session. HCC was diagnosed based on non-invasive criteria in accordance with the guidelines of the European Association for the Study of Liver and American Association for the Study of Liver Disease [22]. The presence of PVTT was confirmed if a low-attenuation intraluminal mass was observed expanding the portal vein or if filling defects were detected in the portal vein branches by three-phase dynamic CT [23].

The inclusion criteria for patients in this study were as follows: (a) age between 18 and 75 years, (b) Eastern Cooperative Oncology Group performance status 0–2, (c) Child-Pugh class A or B liver disease, and (d) presence of PVTT in the right or left portal vein on CT images. Patients were excluded from this study if they (a) had main portal vein obstruction and Child-Pugh class B or C liver disease; (b) had undergone other surgery, ablation treatment, sorafenib therapy, systemic chemotherapy, intra-arterial chemoinfusion, or TACE; (c) had serious medical co-morbidities; or (d) had a history of malignant tumors in addition to HCC. The requirement to obtain informed consent was waived.

### Classification of PVTT

We classified the types of PVTT into three subgroups based on a previous report [18]: (a) Type A was defined as PVTT in the main portal vein; (b) Type B was defined as PVTT in the first-order portal vein branch (the right or left portal vein); (c) Type C was defined as PVTT in the second- or lower-order portal vein branches. All patients with type B PVTT were included in the current study.

### TACE procedure

TACE was performed using a 5-Fr RH catheter or microcatheter (Terumo, Tokyo, Japan) as selectively as possible based on the tumor distribution. Initially, an emulsion of 2–20ml lipiodol (Ultrafluide, Guerbet, France) and 20–40 mg doxorubicin hydrochloride was administered into the feeder vessels. The dosages of lipiodol and doxorubicin were determined according to tumor size, vascularity, presence of arterioportal shunt, and the status of liver function. Next, gelatin sponge particles were administered into the feeder vessels until stasis of arterial flow was achieved. In patients with an arterioportal shunt, embolization with polyvinyl alcohol particles (Polyvinyl Alcohol Foam Embolization Particles; Cook Medical Inc, Bloomington, IN, USA) was performed to occlude the shunt.

### ^125^iodine implantation procedure

**Figure 3:**
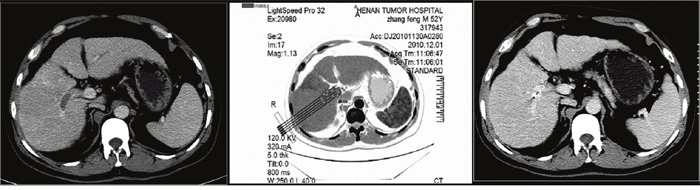
A 43-year-old man with hepatocellular carcinoma complicated with portal vein tumor thrombus in the right branch Enhanced computed tomography showed the portal vein tumor thrombus originated from the right branch of the portal vein. The image prior to iodine seed implantation showed the spatial distribution of seeds according to the treatment planning system. Computed tomography demonstrated that the portal vein tumor thrombus decreased in size and that the portal vein was recanalized two months after seed implantation.

**Figure 4:**
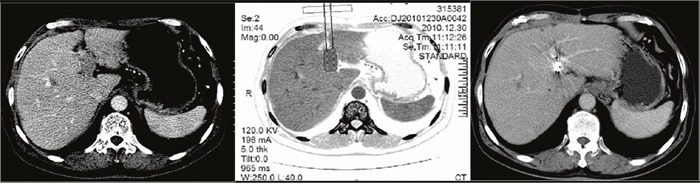
A 65-year-old man with hepatocellular carcinoma complicated with portal vein tumor thrombus in the left branch Enhanced computed tomography showed the thrombus originated from the left branch of the portal vein. The image prior to iodine seed implantation showed the spatial distribution of seeds according to the treatment planning system. Computed tomography demonstrated that the portal vein tumor thrombus decreased in size and that the portal vein was recanalized two months after seed implantation.

**Figure 5:**
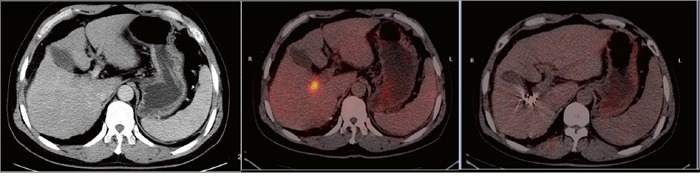
A 45-year-old man with hepatocellular carcinoma complicated with portal vein tumor thrombus in the right branch Enhanced computed tomography showed the thrombus originated from the right branch of the portal vein. Positron emission tomography-computed tomography demonstrated increased fluorodeoxyglucose uptake in the right branch of the portal vein. Positron emission tomography-computed tomography demonstrated no fluorodeoxyglucose uptake in the portal vein tumor thrombus two months after seed implantation.

In the TACE-^125^iodine group, CT-guided ^125^iodine implantation was performed in the PVTT, 4–7 days after the first TACE session. Before implantation, the volume and shape of each PVTT was obtained by CT scanning (Figure 3, Figure 4 and Figure 5) and used to calculate the peripheral dosage. The formula dosage, number, spatial distribution, intensity of radioactivity, and matched peripheral dosage of seeds were then calculated by the treatment planning system (TPS; FTT Technology Ltd. Co, Beijing, China; Figure 3 and Figure 4). The ^125^iodine seeds were implanted under CT guidance in different levels and locations of the portal vein tumor thrombosis. During the procedure, ^125^iodine seeds were implanted into the cancerous embolus 5mm apart along the length and 10mm apart along the width of the PVTT. After the procedure, the needle was retrieved, and the puncture site compressed for hemostasis. All patients underwent a hepatoprotection treatment after seed implantation and before discharge from the hospital.

### Follow-up and repeated TACE or ^125^iodine implantation

All patients who underwent treatment for HCC at our institution were required to undergo follow-up. Each follow-up session included a detailed history and physical examination, laboratory tests, and abdominal contrast material—enhanced three-phase dynamic spiral CT or magnetic resonance (MR) imaging. Laboratory tests included hematological and biochemical analyses, including complete blood cell count; prothrombin time; and α-fetoprotein, aspartate aminotransferase, alanine aminotransferase, total bilirubin, serum albumin, and creatinine levels. All patients were followed up at 4-week intervals after TACE. Patients with an intrahepatic residual viable tumor or recurrent tumor on CT or MR images underwent repeated TACE if the Child-Pugh status remained class A or B. In addition, if residual viable or recurrent PVTT was detected, ^125^iodine seed implantation was repeated. Treatment was terminated if the patient could not tolerate the procedure because of a decline in clinical status, loss to follow-up, or death.

### Patient assessments

The clinical, laboratory, and radiologic records were reviewed. Adverse events of TACE and ^125^iodine implantation were reported according to National Cancer Institute Common Terminology Criteria for Adverse Events, version 3.0 [24]. In the TACE-^125^iodine group, ^125^iodine seed implantation-related adverse events were monitored until the end of seed implantation. In both groups, adverse events that occurred within 4 weeks after TACE were recorded. In addition, liver function tests that were performed 4 weeks after the first ^125^iodine implantation session were used to evaluate treatment toxicity to the liver. Since symptoms of post-embolization syndrome (i.e., abdominal pain, fever without any infection focus, nausea, and vomiting) were expected, they were not documented separately. We compared median overall survival (OS) between TACE-^125^iodine and TACE only patients. OS was defined as the time from the first TACE procedure until death or the last follow-up.

### Statistical analyses

All statistical analyses were performed using SPSS version 22.0 (SPSS, Chicago, IL, USA). To determine significant differences between the two groups, Student's t-test, *χ^2^* test, and the Fisher exact test were used. Kaplan-Meier survival curves were constructed for both groups and evaluated. Univariate analyses were performed using the log rank test. Multivariate analyses was conducted using the Cox proportional hazard regression model on variables with a *P* value < 0.10 in the univariate analyses. The Wilcoxon signed-rank test was used to evaluate the differences in liver function test values before and after treatment. Two tailed statistical tests were performed and *P*<0.05 was considered significant.
